# ExpOmics: a comprehensive web platform empowering biologists with robust multi-omics data analysis capabilities

**DOI:** 10.1093/bioinformatics/btae507

**Published:** 2024-08-10

**Authors:** Douyue Li, Zhuochao Min, Jia Guo, Yubin Chen, Wenliang Zhang

**Affiliations:** The Key Laboratory of Advanced Interdisciplinary Studies, The First Affiliated Hospital of Guangzhou Medical University, GMU-GIBH Joint School of Life Sciences, The Guangdong-Hong Kong-Macao Joint Laboratory for Cell Fate Regulation and Diseases, Guangzhou Medical University, Guangzhou 510182, People’s Republic of China; School of Zoology, The George S. Wise Faculty of Life Sciences, Tel Aviv University, Tel Aviv 6997801, Israel; The Key Laboratory of Advanced Interdisciplinary Studies, The First Affiliated Hospital of Guangzhou Medical University, GMU-GIBH Joint School of Life Sciences, The Guangdong-Hong Kong-Macao Joint Laboratory for Cell Fate Regulation and Diseases, Guangzhou Medical University, Guangzhou 510182, People’s Republic of China; The Key Laboratory of Advanced Interdisciplinary Studies, The First Affiliated Hospital of Guangzhou Medical University, GMU-GIBH Joint School of Life Sciences, The Guangdong-Hong Kong-Macao Joint Laboratory for Cell Fate Regulation and Diseases, Guangzhou Medical University, Guangzhou 510182, People’s Republic of China; The Key Laboratory of Advanced Interdisciplinary Studies, The First Affiliated Hospital of Guangzhou Medical University, GMU-GIBH Joint School of Life Sciences, The Guangdong-Hong Kong-Macao Joint Laboratory for Cell Fate Regulation and Diseases, Guangzhou Medical University, Guangzhou 510182, People’s Republic of China; Department of Hepatobiliary Surgery, The First Affiliated Hospital of Guangzhou Medical University, Guangzhou 510120, People’s Republic of China; Department of Bioinformatics, Outstanding Biotechnology Co., Ltd-Shenzhen, Shenzhen 518026, People’s Republic of China; Department of Pediatrics, The University of Hong Kong-Shenzhen Hospital, Shenzhen 518053, People’s Republic of China

## Abstract

**Motivation:**

High-throughput technologies yield a broad spectrum of multi-omics datasets, which offer unparalleled insights into complex biological systems. However, effectively analyzing this diverse array of data presents challenges, considering factors such as species diversity, data types, costs, and limitations of the available tools.

**Results:**

Herein, we present ExpOmics, a comprehensive web platform featuring 7 applications and 4 toolkits, with 28 customizable analysis functions spanning various analyses of differential expression, co-expression, Weighted Gene Co-expression Network Analysis (WGCNA), feature selection, and functional enrichment. ExpOmics allows users to upload and explore multi-omics data without organism restrictions, supporting various expression data, including genes, mRNAs, lncRNAs, miRNAs, circRNAs, piRNAs, and proteins and is compatible with diverse gene nomenclatures and expression values. Moreover, ExpOmics enables users to analyze 22 427 transcriptomic datasets of 196 cancer subtypes sourced from 63 projects of The Cancer Genome Atlas Program (TCGA) to identify cancer biomarkers. The analysis results from ExpOmics are presented in high-quality graphical formats suitable for publication and are available for free download. A case study using ExpOmics identified two potential oncogenes, *SERPINE1* and *SLC43A1*, that may regulate colorectal cancer through distinct biological processes. In summary, ExpOmics can serves as a robust platform for global researchers to explore multi-omics data, gain biological insights, and formulate testable hypotheses.

**Availability and implementation:**

ExpOmics is available at http://www.biomedical-web.com/expomics.

## 1 Introduction

Multi-omics data generated by high-throughput technologies, such as next-generation sequencing and mass spectrometry, are invaluable resources for understanding complex biological systems (Wang *et al.* 2009, Stark *et al.* 2019). However, despite their potential, mining and analyzing such data remain challenging because of their high costs and thresholds. For instance, the complexity and diversity of multi-omics datasets present a considerable challenge for biologists lacking bioinformatics expertise in exploring these data, including gene, messenger RNA (mRNA), long non-coding RNA (lncRNA), microRNA (miRNA), circular RNA (circRNA), piwi-interacting RNA (piRNA), and protein expression data (Mougin *et al.* 2018). Furthermore, customizable data analysis is often required to address specific biological questions, emphasizing the necessity for user-friendly web platforms to enable the efficient investigation of these complex and diverse data and advance life science research (O'Donoghue *et al.* 2010, Mougin *et al.* 2018). To address these challenges, bioinformaticians have made efforts to develop several web platforms to explore and analyze multi-omics data (Chang *et al.* 2018, Ge *et al.* 2018, Cheng *et al.* 2021, Conard *et al.* 2021, Zhou *et al.* 2021, Liu *et al.* 2022).

However, critical issues remain that need to be urgently addressed. For instance, ExpressVis (Liu *et al.* 2022), eVITTA (Cheng *et al.* 2021), PANDA-view (Chang *et al.* 2018), iDEP (Ge *et al.* 2018), and TIMEOR (Conard *et al.* 2021) primarily focus on gene and protein expression data, catering to specific species, such as Homo sapiens, and model organisms. Consequently, they lack the ability to handle non-coding gene expression data and data from other organisms, including lncRNAs, circRNAs, miRNAs, and piRNAs. Moreover, they lack essential analytical capabilities, such as Weighted Gene Co-expression Network Analysis (WGCNA), feature selection, and integration with large-scale international omics projects, such as The Cancer Genome Atlas (TCGA) (Weinstein *et al.* 2013). Furthermore, they provide relatively limited functionality for differential expression and functional enrichment analyses, and cannot meet the demands of complex analyses.

To fill these gaps, we propose ExpOmics, an easy-to-use web platform featuring applications designed to assist biologists in efficiently processing various types of expression data, without requiring programming skills. ExpOmics offers robust multi-omics data analysis and visualization capabilities for exploring gene, mRNA, lncRNA, miRNA, circRNA, piRNA, and protein expression data, covering various aspects of differential expression, co-expression, WGCNA, feature selection, and functional enrichment analysis. ExpOmics allows users to upload expression data with diverse gene nomenclatures from different resources, and supports various types of expression values and organisms. In addition, ExpOmics integrates 22 427 transcriptomic datasets of 196 cancer subtypes from the TCGA. This integration empowers biologists to thoroughly explore data, thereby facilitating the discovery and validation of cancer biomarkers and targets. In summary, ExpOmics serves as a valuable web platform for equipping biologists worldwide with robust multi-omics data analysis capabilities.

## 2 Materials and methods

### 2.1 Implementation of ExpOmics

**Figure 1.**
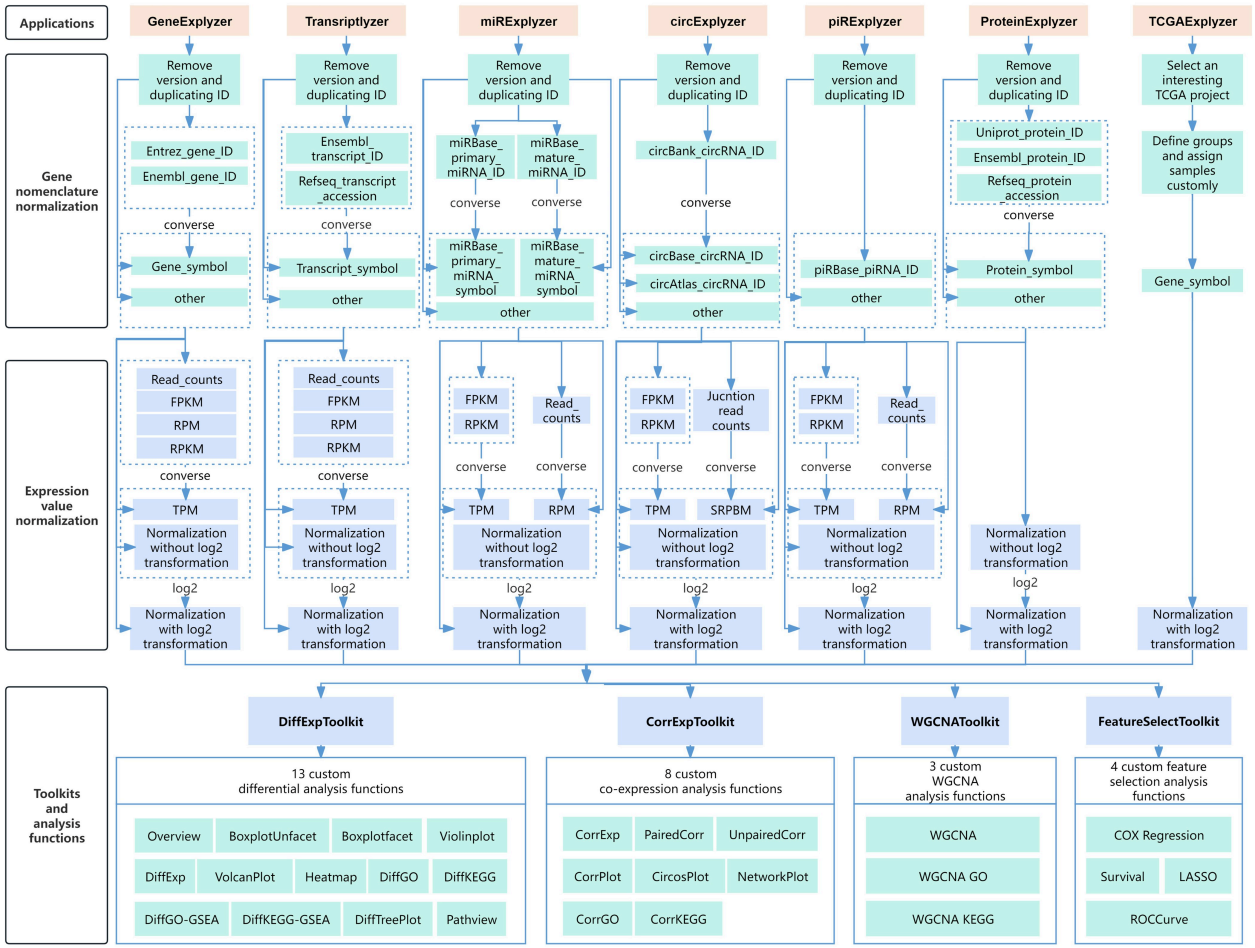
Implementation of ExpOmics.

The ExpOmics web platform was developed using a front-end and back-end separation framework, as previously described (Zhang *et al.* 2022a, Zhang *et al.* 2022b). Within ExpOmics, 7 distinct applications (GeneExplyzer, Transcriptlyzer, miRExplyzer, circExplyzer, piRExplyzer, ProteinExplyzer, and TCGAExplyzer) with four toolkits (DiffExpToolkit, CorrExpToolkit, WGCNAToolkit, and FeatureSelectToolkit) were developed using R (version 4.3.2) (Fig. 1 and Table 1) and seamlessly integrated to provide a comprehensive suite of functionalities (Table 2). The implementation of these applications and toolkits relies on a carefully selected set of R packages and resources, which have been meticulously detailed in Table 1, Supplementary Tables S1 and S2.

**Table 1.** Details of the implementation and utilization of the 7 applications in ExpOmics.

Applications	Description	Data types uploaded by users	Parameters		
			Gene nomenclatures	Types of expression value	Organisms of the expression data source
GeneExplyzer	To upload and standardize gene expression data for custom analysis without organism restriction	Gene expression data and sample metadata	Gene symbol, Entrez gene ID, Ensembl gene ID, and others	Read counts, TPM, RPM, FPKM, RPKM, normalized data with log_2_ transformation, normalized data without log_2_ transformation	*Arabidopsis thaliana, Anopheles gambiae, Bos taurus, Caenorhabditis elegans, Canis familiaris, Drosophila melanogaster, Danio rerio, Gallus gallus, Homo sapiens, Mus musculus, Macaca mulatta, Pan troglodytes, Rattus norvegicus, Saccharomyces cerevisiae, Sus scrofa, Xenopus laevis*, and others
Transcriptlyzer	To upload and standardize mRNA/lncRNA expression data for custom analysis without organism restriction	mRNA/lncRNA expression data and sample metadata	Transcript symbol, RefSeq accession, Ensembl transcript ID, and others		*Arabidopsis thaliana, Bos taurus, Caenorhabditis elegans, Canis familiaris, Drosophila melanogaster, Danio rerio, Gallus gallus, Homo sapiens, Mus musculus, Macaca mulatta, Pan troglodytes, Rattus norvegicus, Saccharomyces cerevisiae, Sus scrofa, Xenopus laevis*, and others
miRExplyzer	To upload and standardize miRNA expression data for custom analysis without organism restriction	miRNA expression data and sample metadata	Primary miRNA ID, mature miRNA ID, primary miRNA symbol, mature miRNA symbol, and others		*Aedes aegypti, Apis mellifera, Arabidopsis thaliana, Bombyx mori, Bos taurus, Caenorhabditis briggsae, Caenorhabditis elegans, Canis familiaris, Chlamydomonas reinhardtii, Danio rerio, Drosophila melanogaster, Drosophila pseudoobscura, Epstein Barr virus, Fugu rubripes, Gallus gallus, Homo sapiens, Human cytomegalovirus, Kaposi sarcoma-associated herpesvirus, Macaca mulatta, Monodelphis domestica, Murid herpesvirus 68, Mus musculus, Oryza sativa, Pan troglodytes, Populus trichocarpa, Rattus norvegicus, Schmidtea mediterranea, Tetraodon nigroviridis, Vitis vinifera, Xenopus tropicalis, Zea mays*, and others
piRExplyzer	To upload and standardize piRNA expression data for custom analysis without organism restriction	piRNA expression data and sample metadata	piRBase ID, piRNA gene ID and symbol in GenBank, and other		*Aplysia californica, Ailuropoda melanoleuca, Biomphalaria glabrata, Bombyx mori, Bos taurus, Caenorhabditis sp. 26 LS-2015, Caenorhabditis sp. 31 LS-2015, Caenorhabditis sp. 32 LS-2015, Caenorhabditis brenneri, Caenorhabditis briggsae, Caenorhabditis castelli, Caenorhabditis doughertyi, Caenorhabditis elegans, Callithrix jacchus, Caenorhabditis remanei, Caenorhabditis virilis, Drosophila erecta, Drosophila melanogaster, Diploscapter pachys, Danio rerio, Drosophila virilis, Drosophila yakuba, Equus caballus, Gallus gallus, Haemonchus contortus, Heligmosomoides polygyrus, Homo sapiens, Mesocricetus auratus, Macaca fascicularis, Macaca mulatta, Mus musculus, Nocardia brasiliensis, Nematostella vectensis, Oryctolagus cuniculus, Oscheius tipulae, Poikilolaimus oxycercus, Pristionchus pacificus, Plectus sambesii, Rattus norvegicus, Scylla paramamosain, Sus scrofa, Tupaia belangeri, Xenopus tropicalis*, and others
circExplyzer	To upload and standardize circRNA expression data for custom analysis without organism restriction	circRNA expression data and sample metadata	circBase ID, circBank ID, circAtlas ID, and others	Read counts, TPM, SRPBM, FPKM, RPKM, normalized data with log_2_ transformation, normalized data without log_2_ transformation	*Canis familiaris, Felis catus, Gallus gallus, Homo sapiens, Mus musculus, Macaca mulatta, Ovis aries, Oryctolagus cuniculus, Rattus norvegicus, Sus scrofa*, and others
ProteinExplyzer	To upload and standardize protein expression data for custom analysis without organism restriction	Protein expression data and sample metadata uploaded by users	Uniprot ID, Ensembl protein ID, and RefSeq protein accession, and others	Normalized data with log_2_ transformation, normalized data without log_2_ transformation	*Arabidopsis thaliana, Anopheles gambiae, Bos taurus, Caenorhabditis elegans, Canis familiaris, Drosophila melanogaster, Danio rerio, Gallus gallus, Homo sapiens, Mus musculus, Macaca mulatta, Pan troglodytes, Rattus norvegicus, Saccharomyces cerevisiae, Sus scrofa, Xenopus laevis*, and others
TCGAExplyzer	For integrated analysis of gene expression data in TCGA		Gene symbol	TPM with log_2_ transformation	*Homo sapiens*

Note: TPM = Transcripts Per Million mapped fragments/reads; RPM = Reads Per Million mapped fragments/reads; FPKM = Fragments Per Kilobase of Million mapped fragments; RPKM = Reads Per Kilobase per Million mapped reads; SRPBM = Spliced Reads Per Billion Mapped fragments/reads.

**Table 2.** Details of the implementation and utilization of the 4 toolkits in ExpOmics.

Toolkit	Description for toolkit	Analysis function	Description for analysis function	Parameters	Outputs
DiffExpToolkit	Providing 13 differential expression analysis functions	Overview	To give an overview of a gene expression data	○ The numbers of top variable gene features: Numbers of top variable gene features for heatmap and PCA	○ A heatmap plot ○ A box plot ○ A PCA plot
		DiffExp	To identify differential expression gene features	○ Log2FC cutoff: A threshold value to define differentially expressed gene feature ○ *P*-value cutoff: A threshold *P*-value to define differentially expressed gene feature	○ A table to show differential expression gene feature
		ViolinPlot	To draw a violin plot	○ Input gene feature(s): one or more gene feature(s) ○ Method: Including t.test, wilcox.test, anova, and kruskal.test ○ *P*-value format: A format of *P*-value for displaying	○ Violin plot(s)
		BoxplotFacet	To draw a box plot with facet	Same to the analysis function of ViolinPlot	○ Facet box plot(s)
		BoxplotUnfacet	To draw a box plot without facet	Same to the analysis function of ViolinPlot	○ Unfacet box plot(s)
		VolcanoPlot	To draw a volcano plot	○ Input gene feature(s): One or more gene feature(s) ○ Log2FC cutoff: A threshold value to define differentially expressed gene feature ○ *P*-value cutoff: A threshold *P*-value to define differentially expressed gene feature	○ A volcano plot
		Heatmap	To draw a heatmap plot	○ Input gene feature(s): At least three gene features ○ Method: Including ward. D, ward. D2, single, complete, average, mcquitty, media, and centroid ○ Cluster column: TRUE or FALSE	○ A heatmap plot
		DiffGO	To perform GO enrichment on differentially expressed gene features	○ Log2FC cutoff: A threshold value to define significant differential gene feature ○ *P*-value cutoff: A threshold *P*-value to define significant differential gene feature ○ Dysregulation type: Including All, Up, and Down ○ GO-*P*-value: A threshold adjust *P*-value for the enriched GO term ○ GO-q-value: A threshold q-value for the enriched GO term ○ Number of terms: A numbers of the GO terms to show	○ A table for the enriched GO terms ○ Two bubble plots ○ A bar plot ○ A chord plot ○ A cluster plot ○ A circle plot
		DiffGO-GSEA	To perform GSEA enrichment on differentially expressed gene features based on GO	Same to the analysis function of DiffGO	○ A table for the enriched GO terms ○ Multiple GSEA plots ○ A ridge plot
		DiffKEGG	To perform KEGG enrichment on differentially expressed gene features	○ Log2FC cutoff: A threshold value to define significant differential gene feature ○ *P*-value cutoff: A threshold *P*-value to define significant differential gene feature ○ Dysregulation type: Including All, Up, and Down ○ KEGG-*P*-value: A threshold *P*-value for the enriched KEGG term ○ KEGG-q-value: A threshold q-value for the enriched KEGG term ○ Number of terms: A numbers of the KEGG terms to show	○ A table for the enriched KEGG terms ○ Two bubble plots ○ A bar plot ○ A chord plot ○ A cluster plot ○ A circle plot
		DiffKEGG-GSEA	To perform GSEA on differentially expressed gene features based on KEGG	Same to the analysis function of DiffKEGG-GSEA	○ A table for the enriched KEGG terms ○ Multiple GSEA plots ○ A ridge plot
		DiffTreePlot	To perform tree plot of GO enrichment on differentially expressed gene features	○ Log2FC cutoff: A threshold value to define significant differential gene feature ○ *P*-value cutoff: A threshold *P*-value to define significant differential gene feature ○ Dysregulation type: Including All, Up, and Down ○ GO-*P*-value: A threshold adjust *P*-value for the enriched GO term ○ GO-q-value: A threshold q-value for the enriched GO term ○ Number of terms: A numbers of the GO terms to show	○ A table for the enriched GO terms ○ A tree plot
		Pathview	To draw a pathview diagram	○ KEGG pathway: A human pathway name in the KEGG resource ○ KEGG native: A parameter to set whether to show the signaling pathway as native	○ A KEGG pathway diagram
CorrExpToolkit	Providing eight co-expression analysis functions	CorrExp	To compute correlationships of a gene feature with others	○ Gene feature: Input a gene feature ○ Correlation cutoff: A threshold value for significant correlation ○ *P*-value cutoff: A threshold *P*-value for significant correlation ○ Method: Including Pearson, Kendall, and Spearman	○ A table for co-expression gene features
		PairedCorr	To draw a paired correlation diagram	○ Gene feature 1: Input gene feature 1 ○ Gene feature 2: Input gene feature 2 ○ Method: Including *t*.test and Wilcox.test	○ A paired box plot
		UnpairedCorr	To draw a linear correlation	○ Gene feature 1: Input gene feature 1 ○ Gene feature 2: Input gene feature 2 ○ Method: Including Pearson, Kendall, and Spearman	○ A correlation linear plot with the scatters
		Corrplot	To draw a corrplot	○ Gene feature(s): Input at least three gene features ○ Method: Including circle, square, ellipse, number, color, pie, and shade	○ A corrplot
		CircosPlot	To draw a circos plot	○ Gene feature(s): Input at least three gene features ○ Method: Including Pearson, Kendall, and Spearman	○ A circos plot
		NetworkPlot	To draw a network plot	○ Gene feature(s): Input at least three gene features ○ Method: Including Pearson, Kendall, and Spearman	○ A network plot
		CorrGO	To identify the biological function of a gene through the GO enrichment of its co-expression genes	○ Gene feature: Input a gene feature ○ Correlation cutoff: A threshold value to define significant correlation ○ Correlation *P*-value cutoff: A threshold *P*-value to define significant correlation ○ GO-*P*-value: A threshold *P*-value for the enriched GO term ○ GO-q-value: A threshold q-value for the enriched GO term ○ GO type: Including All, BC, CC, and MF ○ Number of terms: A numbers of the GO terms to show ○ Method: Including Pearson, Kendall, and Spearman	○ A table for the enriched GO terms ○ Two bubble plots ○ A bar plot
		CorrKEGG	To identify the biological function of a gene through the KEGG enrichment of its co-expression genes	○ Gene feature: Input a gene feature ○ Correlation cutoff: A threshold value to define significant correlation ○ Correlation *P*-value cutoff: A threshold *P*-value to define significant correlation ○ KEGG-*P*-value: A threshold *P*-value for the enriched KEGG term ○ KEGG-q-value: A threshold q-value for the enriched KEGG term ○ Number of terms: A numbers of the KEGG terms to show ○ Method: Including Pearson, Kendall, and Spearman	○ A table for the enriched KEGG terms ○ Two bubble plots ○ A bar plot
WGCNAToolkit	Providing three WGCNA analysis functions	WGCNA	To perform WGCNA, analysis	○ Numbers of gene features: Numbers of top variable gene features ○ Method: A method for sample clustering and module eigengenes ○ MinModule Size: Minimum number of gene features in each module ○ MEDissThres: A threshold to plot the cut line into the dendrogram abline	○ Nine diagrams
		WGCNA GO	To perform GO enrichment on a WGCNA module	○ WGCNA module: A module from WGCNA ○ GO-*P*-value: A threshold *P*-value for the enriched GO term ○ GO-q-value: A threshold q-value for the enriched GO term ○ GO type: Including All, BC, CC, and MF	○ A table for the enriched GO terms ○ A bubble plot ○ A bar plot
		WGCNA KEGG	To perform KEGG pathway enrichment on a WGCNA module	○ WGCNA module: A module from WGCNA ○ KEGG-*P*-value: A threshold *P*-value for the enriched KEGG term ○ KEGG-q-value: A threshold q-value for the enriched KEGG term	○ A table for the enriched KEGG terms ○ A bubble plot ○ A bar plot
FeatureSelectToolkit	Providing four feature selection analysis functions	COX Regression	To draw a forest plot	○ Input a gene feature: A gene feature ○ Expression cutoff: including median, mean, and custom cutoff	○ A forest diagram
		Survival	To draw a Kaplan-Meier survival diagram	○ Input a gene feature: A gene feature ○ Expression cutoff: Including, median, mean, and custom cutoff	○ A survival diagram
		LASSO	To identify risk gene signature	○ Measure method: Including auc, deviance, mse, mae, and class	○ Two tables for gene features ○ A plot of LASSO analysis
		ROCCurve	To dram ROC curves	○ Input gene feature(s): Enter one or more gene feature	○ A plot of ROC curves

Note: BC = biological processes GO terms; CC = cell components GO terms; MF = molecular functions GO terms; GO = Gene Ontology; KEGG = Kyoto Encyclopedia of Genes and Genomes; LASSO = Least Absolute Shrinkage and Selection Operator; ROC = Receiver Operation Curve; TCGA = The Cancer Genome Atlas; WGCNA = Weighted Gene Co-expression Network Analysis.

### 2.2 Implementation of GeneExplyzer and Transcriptlyzer

For GeneExplyzer and Transcriptlyzer, gene annotations spanning 13 organisms were curated from the National Center for Biotechnology Information (NCBI) GenBank (Sayers *et al.* 2024). These annotations included essential details, such as gene symbols, Entrez gene identifiers (IDs), Ensembl gene IDs, transcript symbols, RefSeq accessions, Ensembl transcript IDs, and gene and transcript lengths. By leveraging these rich annotations, we developed GeneExplyzer and Transcriptlyzer applications using R (Fig. 1, Table 1, and Supplementary Table S1).

### 2.3 Implementation of miRExplyzer

To implement miRExplyzer, miRNA annotations spanning 31 organisms were extracted from the miRBase database (Kozomara *et al.* 2019). These annotations included the primary miRNA ID, mature miRNA ID, primary miRNA symbol, mature miRNA symbol, and other relevant details. Subsequently, by leveraging the extracted miRNA annotations, we developed miRExplyzer using R (Fig. 1, Table 1, and Supplementary Table S1).

### 2.4 Implementation of circExplyzer

To implement the circExplyzer, circRNA annotations spanning 10 organisms were extracted from databases, such as circBase (Glazar *et al.* 2014), circBank (Liu *et al.* 2019), and circAtlas (Wu *et al.* 2024). These annotations included circRNA ID, host gene annotations, and other pertinent details. By leveraging this information, circExplyzer was meticulously developed using R (Fig. 1, Table 1, and Supplementary Table S1).

### 2.5 Implementation of piRExplyzer

To implement the piRExplyzer, piRNA annotations spanning 43 organisms were extracted using piRBase (Wang *et al.* 2022). These annotations included piRNA ID, piRNA gene ID, and symbols in GenBank, along with other relevant details. By leveraging the extracted piRNA annotations, we developed piRExplyzer using R (Fig. 1, Table 1, and Supplementary Table S1).

### 2.6 Implementation of ProteinExplyzer

For ProteinExplyzer, the protein annotations of 16 organisms were extracted from UniProt Consortium (2023). These annotations included protein ID and symbols in databases such as UniProt, Ensembl, and RefSeq, along with their host genes and other relevant details. By leveraging this information, the ProteinExplyzer was developed using R (Fig. 1, Table 1, and Supplementary Table S1).

### 2.7 Implementation of TCGAExplyzer

To implement TCGAExplyzer, we extracted and curated gene expression data and sample metadata from TCGA, encompassing 63 projects and 22 427 transcriptomic datasets across 196 cancer subtypes via the Genomic Data Commons (GDC) portal (Weinstein *et al.* 2013) (Table 1). In addition, 4 toolkits featuring 28 analysis functions were developed and incorporated into TCGAExplyzer using R (Fig. 1, Table 1, and Supplementary Table S1).

### 2.8 Implementation of DiffExpToolkit, CorrExpToolkit, WGCNAToolkit, and FeatureSelectToolkit

The 4 toolkits were designed to provide 28 analysis functions for various aspects of differential expression, co-expression, WGCNA, and feature selection analysis, including DiffExpToolkit, CorrExpToolkit, WGCNAToolkit, and FeatureSelectToolkit (Fig. 1 and Table 2). These functions were designed to enable users to select a project, assign samples to predefined groups, and set parameters for custom analysis. A list of the R packages and resources used to implement these toolkits and their analytical functions is provided in Supplementary Table S2.

## 3 Results

### 3.1 Overview of ExpOmics

**Figure 2.**
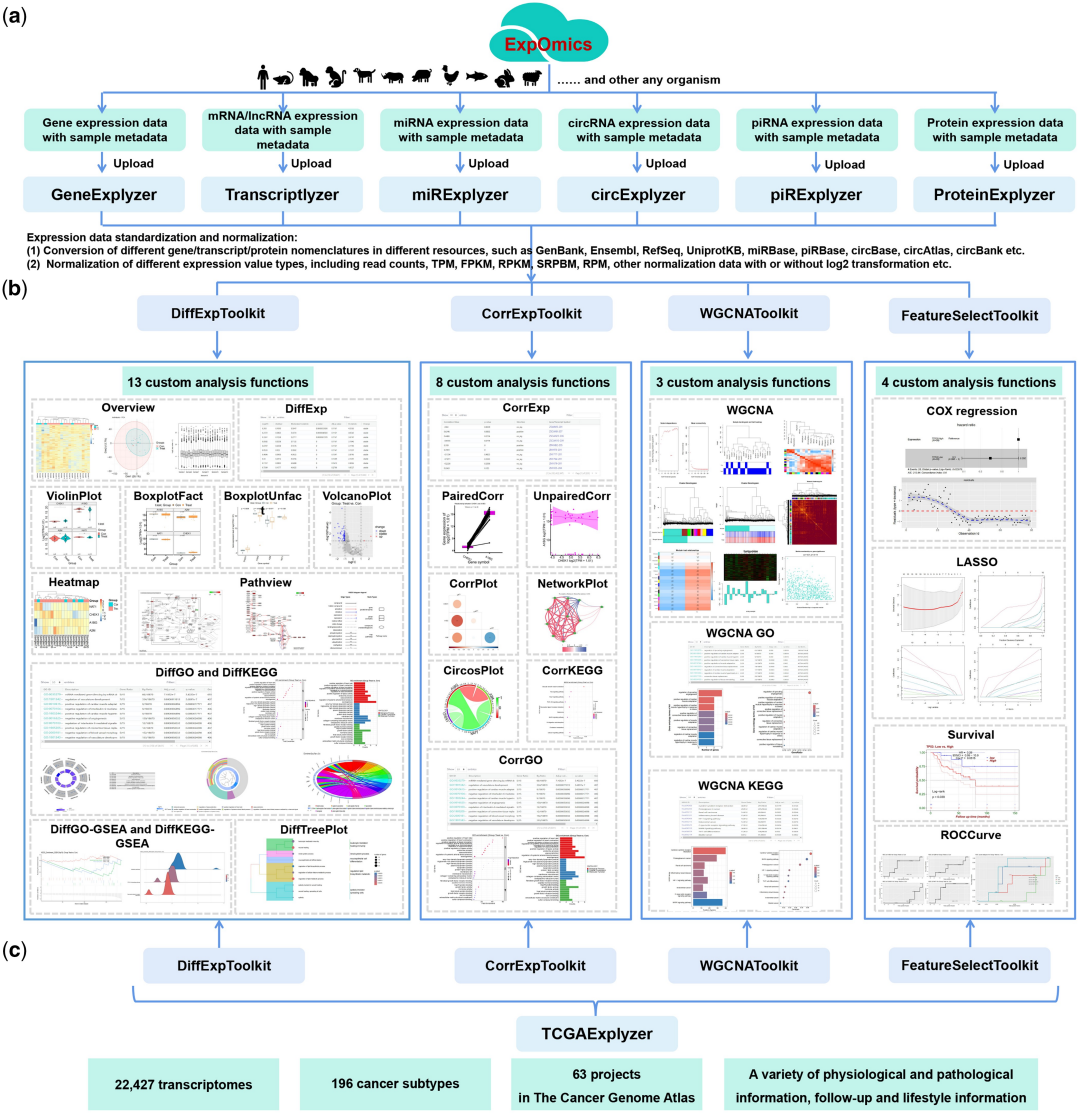
Applications, toolkits, and analysis functions in ExpOmics. Six applications in ExpOmics analyze the expression data of gene, mRNA/lncRNA, miRNA, circRNA, piRNA, respectively (a). Four toolkits perform overall 28 custom analysis functions (b). TCGAExplyzer contains rich TCGA data for analysis (c).

ExpOmics (http://www.biomedical-web.com/expomics) is a user-friendly web platform. It offers 7 meticulously designed applications: GeneExplyzer, Transcriptlyzer, miRExplyzer, circExplyzer, piRExplyzer, ProteinExplyzer, and TCGAExplyzer (Fig. 2a and Table 1). Within these applications, the applications of GeneExplyzer, Transcriptlyzer, miRExplyzer, circExplyzer, piRExplyze, and ProteinExplyzer allow users to upload, standardize, and explore the data of gene, mRNA, lncRNA, miRNA, circRNA, piRNA, and protein expression originating from various organisms (Table 1). Users can specify three parameters, namely organism, gene nomenclature, and expression value type, enabling standardization and analysis across different species with varying gene nomenclature and expression value types (Fig. 2a).

Additionally, users can customize organism and gene nomenclature parameters to “other” for scenarios not covered by default options. Upon completing standardization and data upload, the standardized expression data are stored and assigned a unique identifier for tracking and subsequent analyses. ExpOmics provides a feature called data removal, which allows users to delete their uploaded data using an assigned unique identifier, ensuring data security. Each application was equipped with 4 toolkits with 28 analytical functions for the comprehensive exploration of gene, mRNA, lncRNA, miRNA, circRNA, piRNA, and protein expression data across various organisms (Fig. 2b and Tables 1 and 2). These toolkits include the DiffExpToolkit, CorrExpToolkit, WGCNAToolkit, and FeatureSelectToolkit. Moreover, all these analysis functions enable users to select a project, assign samples to predefined groups, and set parameters for custom analysis based on the sample metadata of the dataset.

In contrast to the GeneExplyzer, Transcriptlyzer, miRExplyzer, circExplyzer, piRExplyzer, and ProteinExplyzer applications, TCGAExplyzer focuses on the comprehensive and custom analysis of 22 427 transcriptomic datasets from 63 projects and 196 cancer subtypes in TCGA (Fig. 2c). Furthermore, the results of these analysis functions are visually presented in high-quality graphical formats suitable for publication and available for free download. Furthermore, the results tables are equipped with filtering and sorting capabilities, allowing users to explore the data easily.

Detailed user guidance and video tutorials are available on the “Help” page and individual application webpages. Extensive tests were conducted across popular web browsers (Google Chrome, Safari, Microsoft Edge, and Firefox) to ensure optimal performance. The details of these applications and toolkits are described below.

### 3.2 Seven applications

#### 3.2.1 GeneExplyzer and Transcriptlyzer

The GeneExplyzer and Transcriptlyzer remove duplicate genes or mRNA/lncRNAs and converts them into standardized gene or transcript symbols in user-uploaded expression data applicable to the listed 13 organisms and others. Moreover, it adeptly normalizes different expression values, including read counts, Reads Per Kilobase per million mapped reads (RPKM), and Fragments Per Kilobase of exon model per Million mapped fragments (FPKM), into Transcripts Per Million (TPM) and seamlessly integrates a log2 transformation process for enhanced analysis.

#### 3.2.2 miRExplyzer

The miRExplyzer aims to remove duplicate miRNA IDs and converts them into standardized miRNA symbols in user-uploaded expression data applicable to the listed 31 organisms and others. Moreover, miRExplyzer normalizes different expression values, such as read counts to Reads Per Million (RPM) with log2 transformation, and adjusts RPKM and FPKM to TPM with log2 transformation.

#### 3.2.3 circExplyzer

The circExplyzer aims to remove duplicate circRNAs and converts them into their standardized circRNA IDs in user-uploaded expression data applicable to the listed 10 organisms and others. Additionally, circExplyzer normalizes different expression values, such as normalizing junction read counts to Spliced Reads Per Billion Mapping (SRPBM) with log2 transformation and adjusting RPKM and FPKM to TPM with log2 transformation.

#### 3.2.4 piRExplyzer

The piRExplyzer aims to remove duplicate piRNAs and converts them into standardized piRNA IDs in user-uploaded expression data applicable to the listed 43 organisms and others. Additionally, piRExplyzer normalizes diverse expression values, such as read counts to RPM with log2 transformation, and RPKM and FPKM to TPM with log2 transformation.

#### 3.2.5 ProteinExplyzer

The ProteinExplyzer aims to remove duplicate proteins and converts them into standardized protein IDs combined with their host gene symbols in user-uploaded expression data for easier recognition, applicable to the listed 16 organisms and others. Additionally, ProteinExplyzer applies a log2 transformation to the uploaded expression data if the transformation was not applied initially.

#### 3.2.6 TCGAExplyzer

The TCGAExplyzer hosts 22 427 transcriptomic datasets from 63 projects and 196 cancer subtypes in TCGA. Moreover, TCGAExplyzer provides 4 toolkits with 28 custom analysis functions for the comprehensive analysis of these data to expedite the discovery of cancer biomarkers and targets.

### 3.3 Four toolkits

#### 3.3.1 DiffExpToolkit

The DiffExpToolkit offers 13 functions for conducting various aspects of the differential expression analysis and visualization of gene, mRNA, lncRNA, miRNA, circRNA, piRNA, and protein expression data from different organisms. These functions include Overview, DiffExp, ViolinPlot, BoxplotFacet, BoxplotUnfacet, VolcanoPlot, Heatmap, DiffGO, DiffGO-GSEA, DiffKEGG, DiffKEGG-GSEA, DiffTreePlot, and Pathview.

#### 3.3.2 CorrExpToolkit

The CorrExpToolkit provides eight co-expression analysis functions for conducting various aspects of co-expression analysis and visualizing gene, mRNA, lncRNA, miRNA, circRNA, piRNA, and protein expression data from different organisms. These functions encompass CorrExp, PairedCorr, UnpairedCorr, CorrPlot, CircosPlot, NetworkPlot, CorrGO, and CorrKEGG.

#### 3.3.3 WGCNAToolkit

The WGCNAToolkit enables users to conduct WGCNA analysis, including network construction, module detection, gene selection, topological property calculations, data simulation, and Gene Ontology (GO) and Kyoto Encyclopedia of Genes and Genomes (KEGG) pathway enrichment analysis on the expression data of genes, mRNAs, lncRNAs, miRNAs, circRNAs, piRNAs, and proteins from different organisms.

#### 3.3.4 FeatureSelectToolkit

The FeatureSelectToolkit provides 4 analysis functions for conducting various aspects of feature selection analysis on the expression data of genes, mRNAs, lncRNAs, miRNAs, circRNAs, piRNAs, and proteins from different organisms to identify and discover important gene features. These functions include COX regression, LASSO, ROCCurve, and Survival.

### 3.4 Case study

**Figure 3.**
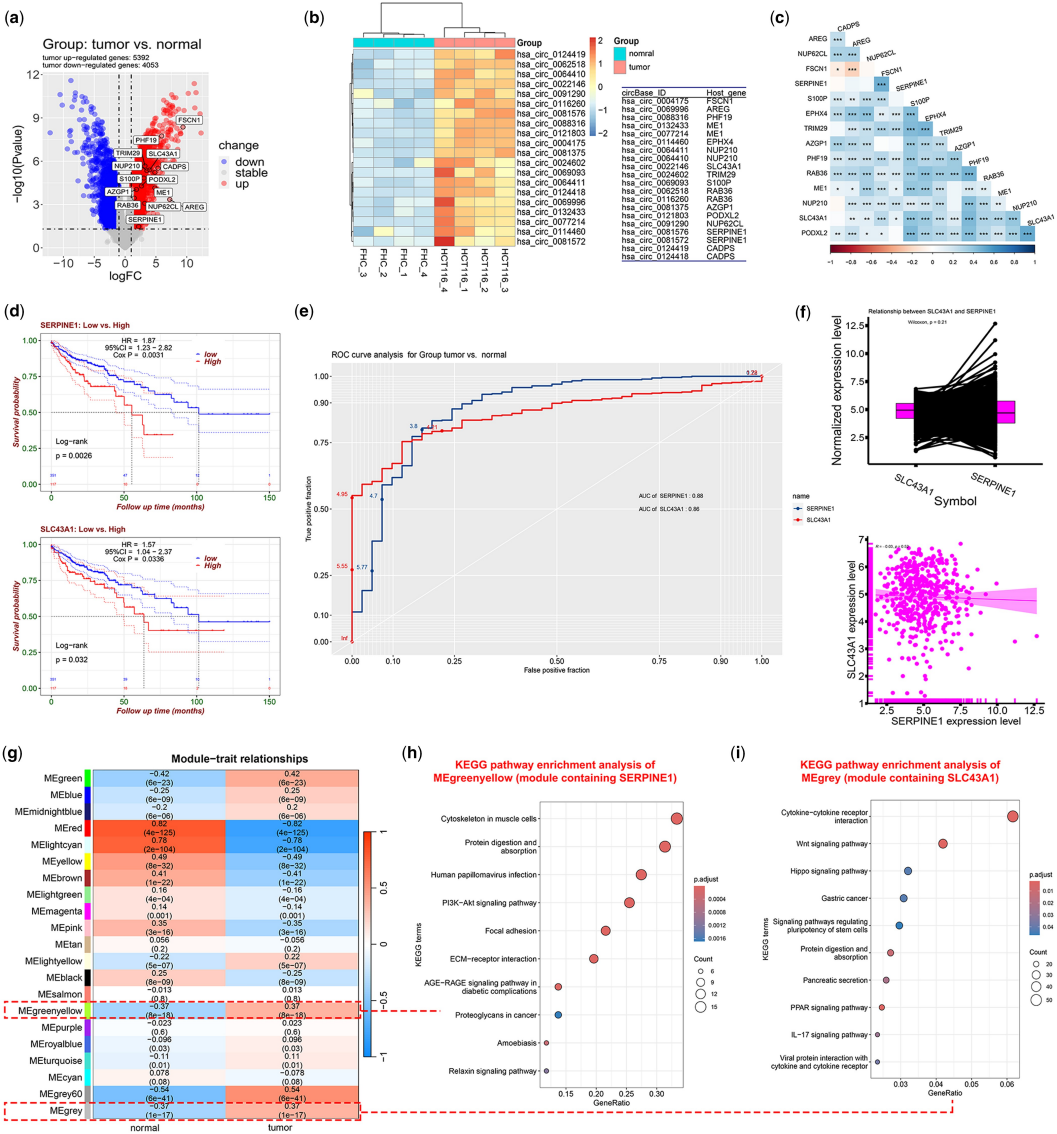
A case study of ExpOmics. The volcano plot (a), heatmap (b), and corrplot (c) of the 15 highly variable genes. The survival (d), ROC (e), correlation (f) analyses of *SERPINE1* and *SLC43A13*. Module-trait relationships from WGCNA analysis (g). KEGG pathway enrichment analyses of the MEgreenyellow (h) and MEgrey (i) modules.

A case study was conducted to demonstrate how this web server benefits biologists. Consequently, a dataset (accession no. GSE211496) from the NCBI Gene Expression Omnibus (GEO) was downloaded and uploaded to ExpOmics for further analyses. This dataset provides the microarray expression profiles of mRNAs, lncRNAs, and circRNAs of human colorectal cancer (CRC). Differential analysis of the GSE211496 and TCGA-COAD datasets identified 15 highly variable genes and their corresponding circRNAs that were upregulated in CRC tumor tissues compared to adjacent tissues (log2 fold-change >1, *P* <.05) (Fig. 3a and b, Supplementary Fig. S1A). Most of the 15 genes showed significant mutual correlations in the correlation analysis of TCGA-COAD dataset (Fig. 3c and Supplementary Fig. S1B).

Moreover, survival analysis indicated that only *SERPINE1* (*PAI-1*) and *SLC43A1* were associated with poor prognosis in patients with CRC (Fig. 3d and Supplementary Fig. S2). Additionally, Receiver Operating Characteristic (ROC) analysis indicated that these genes could independently distinguish between normal adjacent tissues and tumor tissues (Fig. 3e). However, their expression did not correlate in paired or unpaired statistics, suggesting that they may influence CRC development through different molecular pathways (Fig. 3f). Subsequent WGCNA identified multiple co-expression gene modules (Fig. 3g) related to CRC, including MEred, MEyellow, MElightcyan, MEgrey60, MEgreenyellow, and MEgrey.

Further analysis revealed that *SERPINE1* and *SLC43A1* were located in different modules: *SERPINE1* in the MEgreen-yellow module and *SLC43A1* in the MEgrey module. Functional enrichment analysis indicated that genes in the MEgrey module, where *SERPINE1* is located, were mainly associated with the cytoskeleton in muscle cells, focal adhesion, Extracellular Matrix (ECM)-receptor interaction, and the PI3K-Akt signaling pathway (Fig. 3h). In contrast, genes in the MEgreenyellow module, where *SLC43A1* is located, were found to be primarily involved in cytokine–cytokine receptor interactions, the Hippo and WNT signaling pathways, and stem cell pluripotency (Fig. 3i). Consistent with previous studies (Wang *et al.* 2013, Zhao *et al.* 2021, Zhang *et al.* 2022c, Zhao *et al.* 2023, Zhang *et al.* 2024), these results suggest that *SERPINE1* and *SLC43A1* may function as potential oncogenes that regulate CRC through different biological processes.

## 4 Discussion

As demonstrated herein, ExpOmics is an easy-to-use web platform that allows biologists to perform powerful multi-omics data analysis. Compared to existing web platforms, ExpOmics has a wider range of applications and stronger analytical capabilities (Table 3). For instance, compared to mainstream multi-omics web platforms, such as ExpressVis (Liu *et al.* 2022), eVITTA (Cheng *et al.* 2021), and OmicsAnalyst (Zhou *et al.* 2021). ExpOmics supports the expression data of genes, mRNAs, along with proteins and analysis of non-coding RNA expression data, including lncRNAs, circRNAs, miRNAs, and piRNAs. Moreover, ExpOmics can accommodate expression data without species restrictions, whereas ExpressVis and eVITTA only support data from model organisms, such as Homo sapiens, Mus musculus, and Rattus norvegicus. Although OmicsAnalyst (Zhou *et al.* 2021) can display expression data without species restrictions, it lacks the capacity to perform functional enrichment. Both ExpressVis, eVITTA, and OmicsAnalyst also lacks WGCNA functionality, whereas ExpOmics specifically provides this feature. Furthermore, WGCNA reveals intricate relationships between diverse gene features and elucidate how these relationships correlate with phenotypic traits (Langfelder and Horvath 2008). Compared to ExpOmics, their capabilities for differential expression and co-expression analyses are limited, and they have not developed functional modules for the integrated analysis of TCGA data. Altogether, ExpOmics demonstrated superior performance and more comprehensive functionality than similar existing web platforms.

**Table 3.** Comparison of application scope and functionality between ExpOmics and other web platforms.

Web platform name			ExpOmics	ExpressVis	eVITTA	OmicsAnalyst	GEPIA2
Application scope	Gene expression data analysis		√	√	√	√	×
	mRNA/lncRNA expression data analysis		√	√	√	√	×
	miRNA expression data analysis		√	×	×	√	×
	circRNA expression data analysis		√	×	×	×	×
	piRNA expression data analysis		√	×	×	×	×
	Protein expression data analysis		√	×	√	√	×
	TCGA expression data analysis		√	×	×	×	√
Functionality	DiffExpToolkit	Overview	√	×	×	√	√
		Differential expression analysis	√	√	√	×	√
		Violin plot	√	×	√	×	√
		Boxplot unfacet	√	×	×	×	√
		Boxplot facet	√	√	×	√	×
		Volcano plot	√	√	√	√	×
		Heatmap	√	√	√	×	√
		GO enrichment analysis	√	√	×	×	×
		GO GSEA analysis	√	√	√	×	×
		KEGG enrichment analysis	√	√	×	×	×
		KEGG GSEA analysis	√	√	√	×	×
		GO tree plot	√	×	× (Gene set tree)	×	×
		Pathview	√	√	√	×	
	CorrExpToolkit	Co-expression analysis	√	√	√	√	√
		Paired correlation analysis	√	×	×	×	×
		Unpaired correlation analysis	√	√	√	×	√
		Corrplot analysis	√	×	×	×	×
		Circosplot analysis	√	×	×	×	×
		Networkplot analysis	√	×	×	√	×
		GO enrichment analysis	√	×	×	×	×
		KEGG enrichment analysis	√	×	×	×	×
	WGCNAToolkit	WGCNA analysis	√	×	×	×	×
		GO enrichment analysis	√	×	×	×	×
		KEGG enrichment analysis	√	×	×	×	×
	FeatureSelectToolkit	COX Regression analysis	√	×	×	×	×
		Survival analysis	√	√	×	×	√
		LASSO analysis	√	×	×	×	×
		ROC analysis	√	×	×	×	×
	PPI network	PPI Network analysis	×	√	×	×	×
	PTM analysis	PTM analysis	×	×	×	×	×
Without organism restriction			√	× (Human, mouse, and rat)	× (Human and model organism)	√	× (Human)
Custom design for assigning specific samples to defined groups			√	√	√	×	×
Allowing upload data for analysis			√	√	√	√	×

Note: “-,” the data and function feature is not provided or cannot be statistically calculated; “√,” the data and function feature is provided by the database; “×,” the data and function feature is not provided by the database; GO = Gene Ontology; KEGG = Kyoto Encyclopedia of Genes and Genomes; LASSO = Least Absolute Shrinkage and Selection Operator; ROC = Receiver Operating Characteristic; WGCNA = Weighted Gene Co-expression Network Analysis; PPI = Protein–Protein Interaction.

## 5 Conclusion

In the foreseeable future, we plan to enhance ProteinExplyzer in ExpOmics by integrating a protein–protein interaction network analysis function. The web platform will be updated to adapt expression data at larger scales, such as single-cell and spatial transcriptomes, and more custom parameters, such as plot size, plot color scheme, user-defined gene matrix transposed file, and NCBI GEO platform files. We are committed to the ongoing maintenance and improvement of ExpOmics regarding analytical and visualization capabilities, and we expect these updates to significantly boost the utility of ExpOmics in the analysis of multi-omics data.

## Supplementary Material

btae507_Supplementary_Data

## Data Availability

ExpOmics is available at http://www.biomedical-web.com/expomics. The web platform is freely accessible without a login requirement.

## References

[btae507-B1] Chang C , Xu K, Guo C et al PANDA-view: an easy-to-use tool for statistical analysis and visualization of quantitative proteomics data. Bioinformatics 2018;34:3594–6. 29790911 10.1093/bioinformatics/bty408 PMC6184437

[btae507-B2] Cheng X , Yan J, Liu Y et al eVITTA: a web-based visualization and inference toolbox for transcriptome analysis. Nucleic Acids Res 2021;49:W207–W215. 34019643 10.1093/nar/gkab366 PMC8218201

[btae507-B3] Conard AM , Goodman N, Hu Y et al TIMEOR: a web-based tool to uncover temporal regulatory mechanisms from multi-omics data. Nucleic Acids Res 2021;49:W641–W653. 34125906 10.1093/nar/gkab384 PMC8262710

[btae507-B4] Ge SX , Son EW, Yao R. iDEP: an integrated web application for differential expression and pathway analysis of RNA-Seq data. BMC Bioinformatics 2018;19:534. 30567491 10.1186/s12859-018-2486-6 PMC6299935

[btae507-B5] Glazar P , Papavasileiou P, Rajewsky N. circBase: a database for circular RNAs. RNA 2014;20:1666–70. 25234927 10.1261/rna.043687.113 PMC4201819

[btae507-B6] Kozomara A , Birgaoanu M, Griffiths-Jones S. miRBase: from microRNA sequences to function. Nucleic Acids Res 2019;47:D155–D162. 30423142 10.1093/nar/gky1141 PMC6323917

[btae507-B7] Langfelder P , Horvath S. WGCNA: an R package for weighted correlation network analysis. BMC Bioinformatics 2008;9:559. 19114008 10.1186/1471-2105-9-559 PMC2631488

[btae507-B8] Liu M , Wang Q, Shen J et al circBank: a comprehensive database for circRNA with standard nomenclature. RNA Biol 2019;16:899–905. 31023147 10.1080/15476286.2019.1600395 PMC6546381

[btae507-B9] Liu X , Xu K, Tao X et al ExpressVis: a biologist-oriented interactive web server for exploring multi-omics data. Nucleic Acids Res 2022;50:W312–W321. 35639516 10.1093/nar/gkac399 PMC9252728

[btae507-B10] Mougin F , Auber D, Bourqui R et al Visualizing omics and clinical data: which challenges for dealing with their variety? Methods 2018;132:3–18. 28887085 10.1016/j.ymeth.2017.08.012

[btae507-B11] O'Donoghue SI , Gavin AC, Gehlenborg N et al Visualizing biological data-now and in the future. Nat Methods 2010;7:S2–4. 20195254 10.1038/nmeth.f.301

[btae507-B12] Sayers EW , Cavanaugh M, Clark K et al GenBank 2024 update. Nucleic Acids Res 2024;52:D134–D137. 37889039 10.1093/nar/gkad903 PMC10767886

[btae507-B13] Stark R , Grzelak M, Hadfield J. RNA sequencing: the teenage years. Nat Rev Genet 2019;20:631–56. 31341269 10.1038/s41576-019-0150-2

[btae507-B14] UniProt Consortium. UniProt: the universal protein knowledgebase in 2023. Nucleic Acids Res 2023;51:D523–D531. 36408920 10.1093/nar/gkac1052 PMC9825514

[btae507-B15] Wang Z , Gerstein M, Snyder M. RNA-Seq: a revolutionary tool for transcriptomics. Nat Rev Genet 2009;10:57–63. 19015660 10.1038/nrg2484 PMC2949280

[btae507-B16] Wang J , Shi Y, Zhou H et al piRBase: integrating piRNA annotation in all aspects. Nucleic Acids Res 2022;50:D265–D272. 34871445 10.1093/nar/gkab1012 PMC8728152

[btae507-B17] Wang Q , Tiffen J, Bailey CG et al Targeting amino acid transport in metastatic castration-resistant prostate cancer: effects on cell cycle, cell growth, and tumor development. J Natl Cancer Inst 2013;105:1463–73. 24052624 10.1093/jnci/djt241

[btae507-B18] Weinstein JN , Collisson EA, Mills GB et al; Cancer Genome Atlas Research Network. The cancer genome atlas pan-cancer analysis project. Nat Genet 2013;45:1113–20. 24071849 10.1038/ng.2764 PMC3919969

[btae507-B19] Wu W , Zhao F, Zhang J. circAtlas 3.0: a gateway to 3 million curated vertebrate circular RNAs based on a standardized nomenclature scheme. Nucleic Acids Res 2024;52:D52–D60. 37739414 10.1093/nar/gkad770 PMC10767913

[btae507-B20] Zhang W , Liu Y, Min Z et al circMine: a comprehensive database to integrate, analyze and visualize human disease-related circRNA transcriptome. Nucleic Acids Res2022a;50:D83–D92. 34530446 10.1093/nar/gkab809 PMC8728235

[btae507-B21] Zhang W , Yang S, Chen D et al SOX_2_-OT induced by PAI-1 promotes triple-negative breast cancer cells metastasis by sponging miR-942-5p and activating PI3K/Akt signaling. Cell Mol Life Sci2022c;79:59. 34997317 10.1007/s00018-021-04120-1 PMC11072091

[btae507-B22] Zhang W , Zhang Y, Min Z et al COVID19db: a comprehensive database platform to discover potential drugs and targets of COVID-19 at whole transcriptomic scale. Nucleic Acids Res2022b;50:D747–D757. 34554255 10.1093/nar/gkab850 PMC8728200

[btae507-B23] Zhang D , Zhang JW, Xu H et al Therapy-induced senescent tumor cell-derived extracellular vesicles promote colorectal cancer progression through SERPINE1-mediated NF-kappaB p65 nuclear translocation. Mol Cancer 2024;23:70. 38576002 10.1186/s12943-024-01985-1 PMC10993572

[btae507-B24] Zhao X , Sakamoto S, Wei J et al Contribution of the l-type amino acid transporter family in the diagnosis and treatment of prostate cancer. Int J Mol Sci 2023;24:6178. 37047148 10.3390/ijms24076178 PMC10094571

[btae507-B25] Zhao Q , Xie J, Xie J et al Weighted correlation network analysis identifies FN1, COL1A1 and SERPINE1 associated with the progression and prognosis of gastric cancer. Cancer Biomark 2021;31:59–75. 33780362 10.3233/CBM-200594 PMC12499999

[btae507-B26] Zhou G , Ewald J, Xia J. OmicsAnalyst: a comprehensive web-based platform for visual analytics of multi-omics data. Nucleic Acids Res 2021;49:W476–W482. 34019646 10.1093/nar/gkab394 PMC8262745

